# Supplementary material for: Identification of putative causal loci in whole-genome sequencing data via knockoff statistics
Source: Nat Commun. 2021 May 25;12:3152. doi: 10.1038/s41467-021-22889-4 (PMC8149672; doi:10.1038/s41467-021-22889-4)
Supplement: Supplementary file 1 — Supplementary Information [file 41467_2021_22889_MOESM1_ESM.pdf]

Identification of putative causal loci in whole-genome sequencing data via knockoff statistics

He et al.

Supplementary Information for "Identification of putative causal loci in whole-genome sequencing data via knockoff statistics"

Zihuai He[1,2#], Linxi Liu[3], Chen Wang[4], Yann Le Guen[1], Justin Lee[2], Stephanie Gogarten[5], Fred Lu[6], Stephen Montgomery[7,8], Hua Tang[6,7], Edwin K. Silverman[9], Michael H. Cho[9], Michael Greicius[1], Iuliana Ionita-Laza[4#]

[1]Department of Neurology and Neurological Sciences, Stanford University, Stanford, CA 94305, USA
[2]Quantitative Sciences Unit, Department of Medicine, Stanford University, Stanford, CA, 94305, USA
[3]Department of Statistics, Columbia University, New York, NY 10027, USA
[4]Department of Biostatistics, Columbia University, New York, NY 10032, USA
[5]Department of Biostatistics, University of Washington, Seattle, WA, USA
[6]Department of Statistics, Stanford University, Stanford, CA, 94305, USA
[7]Department of Genetics, Stanford University, Stanford, CA, 94305, USA
[8]Department of Pathology, Stanford University, Stanford, CA, 94305, USA
[9]Channing Division of Network Medicine and Division of Pulmonary and Critical Care Medicine Division, Brigham and Women's Hospital, Harvard Medical School, Boston, MA, 02215, USA

#Correspondence to: Zihuai He (zihuai@stanford.edu) and Iuliana Ionita-Laza (ii2135@cumc.columbia.edu)

Supplementary Note

Additional acknowledgement

The Alzheimer's Disease Sequencing Project (ADSP) is comprised of two Alzheimer's Disease (AD) genetics consortia and three National Human Genome Research Institute (NHGRI) funded Large Scale Sequencing and Analysis Centers (LSAC). The two AD genetics consortia are the Alzheimer's Disease Genetics Consortium (ADGC) funded by NIA (U01 AG032984), and the Cohorts for Heart and Aging Research in Genomic Epidemiology (CHARGE) funded by NIA (R01 AG033193), the National Heart, Lung, and Blood Institute (NHLBI), other National Institute of Health (NIH) institutes and other foreign governmental and non-governmental organizations. The Discovery Phase analysis of sequence data is supported through UF1AG047133 (to Drs. Schellenberg, Farrer, Pericak-Vance, Mayeux, and Haines); U01AG049505 to Dr. Seshadri; U01AG049506 to Dr. Boerwinkle; U01AG049507 to Dr. Wijsman; and U01AG049508 to Dr. Goate and the Discovery Extension Phase analysis is supported through U01AG052411 to Dr. Goate, U01AG052410 to Dr. Pericak-Vance and U01 AG052409 to Drs. Seshadri and Fornage. Data generation and harmonization in the Follow-up Phases is supported by U54AG052427 (to Drs. Schellenberg and Wang).

The ADGC cohorts include: Adult Changes in Thought (ACT), the Alzheimer's Disease Centers (ADC), the Chicago Health and Aging Project (CHAP), the Memory and Aging Project (MAP), Mayo Clinic (MAYO), Mayo Parkinson's Disease controls, University of Miami, the Multi-Institutional Research in Alzheimer's Genetic Epidemiology Study (MIRAGE), the National Cell Repository for Alzheimer's Disease (NCRAD), the National Institute on Aging Late Onset Alzheimer's Disease Family Study (NIA-LOAD), the Religious Orders Study (ROS), the Texas Alzheimer's Research and Care Consortium (TARC), Vanderbilt University/Case Western Reserve University (VAN/CWRU), the Washington Heights-Inwood Columbia Aging Project (WHICAP) and the Washington University Sequencing Project (WUSP), the Columbia University Hispanic- Estudio Familiar de Influencia Genetica de Alzheimer (EFIGA), the University of Toronto (UT), and Genetic Differences (GD).

The CHARGE cohorts are supported in part by National Heart, Lung, and Blood Institute (NHLBI) infrastructure grant HL105756 (Psaty), RC2HL102419 (Boerwinkle) and the neurology working group is supported by the National Institute on Aging (NIA) R01 grant AG033193. The CHARGE cohorts participating in the ADSP include the following: Austrian Stroke Prevention Study (ASPS), ASPS-Family study, and the Prospective Dementia Registry-Austria (ASPS/PRODEM-Aus), the Atherosclerosis Risk in Communities (ARIC) Study, the Cardiovascular Health Study (CHS), the Erasmus Rucphen Family Study (ERF), the Framingham Heart Study (FHS), and the Rotterdam Study (RS). ASPS is funded by the Austrian Science Fond (FWF) grant number P20545-P05 and P13180 and the Medical University of Graz. The ASPS-Fam is funded by the Austrian Science Fund (FWF) project I904),the EU Joint Programme - Neurodegenerative Disease Research (JPND) in frame of the BRIDGET project (Austria, Ministry of Science) and the Medical University of Graz and the Steiermärkische Krankenanstalten Gesellschaft. PRODEM-Austria is supported by the Austrian Research Promotion agency (FFG) (Project No. 827462) and by the Austrian National Bank (Anniversary Fund, project 15435. ARIC research is carried out as a collaborative study supported by NHLBI contracts (HHSN268201100005C, HHSN268201100006C, HHSN268201100007C, HHSN268201100008C, HHSN268201100009C, HHSN268201100010C, HHSN268201100011C, and HHSN268201100012C). Neurocognitive data in ARIC is collected by U01 2U01HL096812, 2U01HL096814, 2U01HL096899, 2U01HL096902, 2U01HL096917 from the NIH (NHLBI, NINDS, NIA and NIDCD), and with previous brain MRI examinations funded by R01-HL70825 from the NHLBI. CHS research was supported by contracts HHSN268201200036C, HHSN268200800007C, N01HC55222, N01HC85079, N01HC85080, N01HC85081, N01HC85082, N01HC85083, N01HC85086, and grants U01HL080295 and U01HL130114 from the NHLBI with additional contribution from the National Institute of Neurological Disorders and Stroke (NINDS). Additional support was provided by R01AG023629, R01AG15928, and R01AG20098 from the NIA. FHS research is supported by NHLBI contracts N01-HC-25195 and HHSN268201500001I. This study was also

supported by additional grants from the NIA (R01s AG054076, AG049607 and AG033040 and NINDS (R01 NS017950). The ERF study as a part of EUROSPAN (European Special Populations Research Network) was supported by European Commission FP6 STRP grant number 018947 (LSHG-CT-2006-01947) and also received funding from the European Community's Seventh Framework Programme (FP7/2007-2013)/grant agreement HEALTH-F4-2007-201413 by the European Commission under the programme "Quality of Life and Management of the Living Resources" of 5th Framework Programme (no. QLG2-CT-2002-01254). High-throughput analysis of the ERF data was supported by a joint grant from the Netherlands Organization for Scientific Research and the Russian Foundation for Basic Research (NWO-RFBR 047.017.043). The Rotterdam Study is funded by Erasmus Medical Center and Erasmus University, Rotterdam, the Netherlands Organization for Health Research and Development (ZonMw), the Research Institute for Diseases in the Elderly (RIDE), the Ministry of Education, Culture and Science, the Ministry for Health, Welfare and Sports, the European Commission (DG XII), and the municipality of Rotterdam. Genetic data sets are also supported by the Netherlands Organization of Scientific Research NWO Investments (175.010.2005.011, 911-03-012), the Genetic Laboratory of the Department of Internal Medicine, Erasmus MC, the Research Institute for Diseases in the Elderly (014-93-015; RIDE2), and the Netherlands Genomics Initiative (NGI)/Netherlands Organization for Scientific Research (NWO) Netherlands Consortium for Healthy Aging (NCHA), project 050-060-810. All studies are grateful to their participants, faculty and staff. The content of these manuscripts is solely the responsibility of the authors and does not necessarily represent the official views of the National Institutes of Health or the U.S. Department of Health and Human Services.

The four LSACs are: the Human Genome Sequencing Center at the Baylor College of Medicine (U54 HG003273), the Broad Institute Genome Center (U54HG003067), The American Genome Center at the Uniformed Services University of the Health Sciences (U01AG057659), and the Washington University Genome Institute (U54HG003079).

Biological samples and associated phenotypic data used in primary data analyses were stored at Study Investigators institutions, and at the National Cell Repository for Alzheimer’s Disease (NCRAD, U24AG021886) at Indiana University funded by NIA. Associated Phenotypic Data used in primary and secondary data analyses were provided by Study Investigators, the NIA funded Alzheimer’s Disease Centers (ADCs), and the National Alzheimer’s Coordinating Center (NACC, U01AG016976) and the National Institute on Aging Genetics of Alzheimer’s Disease Data Storage Site (NIAGADS, U24AG041689) at the University of Pennsylvania, funded by NIA, and at the Database for Genotypes and Phenotypes (dbGaP) funded by NIH. This research was supported in part by the Intramural Research Program of the National Institutes of health, National Library of Medicine. Contributors to the Genetic Analysis Data included Study Investigators on projects that were individually funded by NIA, and other NIH institutes, and by private U.S. organizations, or foreign governmental or nongovernmental organizations.

The COPDGene project was supported by Award Number U01 HL089897 and Award Number U01 HL089856 from the National Heart, Lung, and Blood Institute. The content is solely the responsibility of the authors and does not necessarily represent the official views of the National Heart, Lung, and Blood Institute or the National Institutes of Health. COPDGene is also supported by the COPD Foundation through contributions made to an Industry Advisory Board comprised of AstraZeneca, Boehringer-Ingelheim, Genentech, GlaxoSmithKline, Novartis, Pfizer, Siemens, and Sunovion.

Molecular data for the Trans-Omics in Precision Medicine (TOPMed) program was supported by the National Heart, Lung and Blood Institute (NHLBI). Genome Sequencing for "NHLBI TOPMed: Genetic Epidemiology of COPD (COPDGene) in the TOPMed Program” (phs000951) was performed at the University of Washington Northwest Genomics Center (3R01 HL089856-08S1) and the Broad Institute of MIT and Harvard (HHSN268201500014C). Core support including centralized genomic read mapping and genotype calling, along with variant quality metrics and filtering were provided by the TOPMed Informatics Research Center (3R01HL-117626-02S1; contract HHSN268201800002I). Core support including

phenotype harmonization, data management, sample-identity QC, and general program coordination were provided by the TOPMed Data Coordinating Center (R01HL-120393; U01HL-120393; contract HHSN268201800001I). We gratefully acknowledge the studies and participants who provided biological samples and data for TOPMed.

The probabilistic model for genetic variables. As a proof of concept, we demonstrate the exchangeability of the proposed sequential knockoff generator for multivariate Gaussian distribution. The application of the proposed method to genotype dosage data is an approximation, and we demonstrate the practical performance of the proposed method by empirical studies.

The knockoff generator introduced in the paper is proposed based on a multivariate Gaussian approximation. Specifically, let $G = (G_1, \cdots, G_p)^T$ be the collection of p genetic variants. We assume a multivariate normal model for G: $G \sim N(\mu, \Sigma)$. Based on the known haplotype block structure in the human genome, we also assume the covariance matrix Σ is block diagonal, i.e.,

$$\Sigma = \begin{bmatrix} \Sigma_{11} & & 0 \\ & \ddots & \\ 0 & & \Sigma_{LL} \end{bmatrix},$$

where each Σ_{ll} $(1 \leq l \leq L)$ is a k_l by k_l matrix. This is to say, if we divide the genome into L $(L \leq p)$ contiguous non-overlapping regions/blocks, and use $\Phi_1, \cdots, \Phi_L$ to denote the set of genetic variants contained in each region respectively and let $k_l = |\Phi_l|$ $(1 \leq l \leq L)$, then with appropriately spaced and sized regions, we may use a model in which the variants from different regions are independent to each other as an approximation to the underlying correlations structure. Let $\Theta = \Sigma^{-1}$ be the precision matrix. It is easy to see that Θ is also a block diagonal matrix. For each variant j, let $B_j = \{j' \in [p], j' \neq j : \Theta_{jj'} \neq 0\}$. Then conditional on the variants $\{G_{j'}, j' \in B_j\}$, the variant G_j is independent of the other variants. Based on our assumption, if $j \in \Phi_l$ for some l, then $B_j \subset \Phi_l$.

We also introduce the following notation: if G is a vector of p random variables, for any $A \subset [p]$, G_A is defined to be the column vector $(G_j)_{j \in A}$.

When the model parameters are known, we claim that if we apply the Algorithm 1 Sequential Conditional Independent Pairs (Single Knockoff) to this model, the following claims hold at each step j $(1 \leq j \leq p)$:

1. When sample $\tilde{G}_j$ from $\mathcal{L}(G_j | G_{-j}, \tilde{G}_{1:(j-1)})$ (with the convention that for $j = 1$, we sample from $\mathcal{L}(G_j | G_{-j})$), it becomes sampling $\tilde{G}_j$ from $N(\tilde{\mu}_j, \tilde{\sigma}_j^2)$, where $\tilde{\mu}_j$ is a linear combination of variants G_{Φ_l} and $\tilde{G}_{\Phi_l \cap [j-1]}$ if $j \in \Phi_l$ (for $j = 1$, it is only G_{Φ_l}).
2. $(G, \tilde{G}_{1:j})$ jointly follow a multivariate Gaussian distribution, and if we denote the precision matrix of this distribution by $\Theta^{(j+p)}$, then for any $s \in \Phi_l, t \in \Phi_m$ with $l \neq m$, $\Theta^{(j+p)}_{st} = 0$, $\Theta^{(j+p)}_{s,(t+p)} = 0$ when $t \leq$j, $\Theta^{(j+p)}_{(s+p),t} = 0$ when $s \leq$j, and $\Theta^{(j+p)}_{(s+p),(t+p)}$=0, when both $s \leq$j and $t \leq$j.

The claims can be shown by induction. It is easy to see that the claims hold when $j = 1$. Assume the claims hold up to step j. Then at step $j + 1$, as $(G, \tilde{G}_{1:j})$ follows a multivariate normal distribution, the conditional distribution of G_{j+1} given $(G_{-(j+1)}, \tilde{G}_{1:j})$ is again a normal one. If we denote the mean of this conditional distribution as $\tilde{\mu}_{(j+1)}$, then $\tilde{\mu}_{(j+1)}$ should be a linear function of $G_{B^{(j+p)}_{j+1}}$ with $B^{(j+p)}_{j+1} = \{j' \neq j + 1: \Theta^{(j+p)}_{j+1,j'} \neq 0, 1 \leq j' \leq p\}$ and $\tilde{G}_{\tilde{B}^{(j+p)}_{j+1}}$ with $\tilde{B}^{(j+p)}_{j+1} = \{j' - p: \Theta^{(j+p)}_{j+1,j'} \neq 0, p < j' \leq p + j\}$. Based on the second induction hypothesis at step j, if $j + 1 \in \Phi_l$ for some l, then $G_{B^{(j+p)}_{j+1}} \subset \Phi_l$ and $\tilde{G}_{\tilde{B}^{(j+p)}_{j+1}} \subset \Phi_l \cap [j]$. Therefore, the first induction hypothesis still holds at step $j + 1$.

To show the second part, without loss of generality, we can switch the order of the variables to make G_{j+1} the last variable, and the corresponding precision matrix is denoted as

$$\overline{\Theta}^{(j+p)} = \begin{bmatrix} \overline{\Theta}_1^{(j+p)} & \bar{\theta}_{j+1} \\ \bar{\theta}_{j+1}^T & \Theta_{j+1,j+1}^{(j+p)} \end{bmatrix},$$

where $\overline{\Theta}_1^{(j+p)}$ is a $p+j-1$ by $p+j-1$ matrix obtained by removing the $(j+1)$st column and $(j+1)$st row from $\Theta^{(j+p)}$, $\bar{\theta}_{j+1}$ is a column vector of length $p+j-1$, obtained by removing the $(j+1)$st element from the $(j+1)$st column of $\Theta^{(j+p)}$, and $\Theta_{j+1,j+1}^{(j+p)}$ is the $(j+1)$st diagonal element of $\Theta^{(j+p)}$. Then after we sample $\tilde{G}_{j+1}$ independently from $\mathcal{L}\left(G_{j+1} \middle| G_{-(j+1)}, \tilde{G}_{1:j}\right)$, the joint distribution of $\left(G_{-(j+1)}, \tilde{G}_{1:j}, G_{j+1}, \tilde{G}_{j+1}\right)$ is still a multivariate normal one, and its precision matrix is

$$\overline{\Theta}^{(j+p+1)} = \begin{bmatrix} \overline{\Theta}_1^{(j+p)} + \left(\Theta_{j+1,j+1}^{(j+p)}\right)^{-1} \bar{\theta}_{j+1}\bar{\theta}_{j+1}^T & \bar{\theta}_{j+1} & \bar{\theta}_{j+1} \\ \bar{\theta}_{j+1}^T & \Theta_{j+1,j+1}^{(j+p)} & 0 \\ \bar{\theta}_{j+1}^T & 0 & \Theta_{j+1,j+1}^{(j+p)} \end{bmatrix}.$$

Based on this, after rearrange the order of variables, we can get the precision matrix of the joint distribution of $\left(G, \tilde{G}_{1:(j+1)}\right)$, which is denoted as $\Theta^{(j+p+1)}$. Based on the second induction hypothesis, we still have for any $s \in \Phi_l$, $t \in \Phi_m$ with $l \neq m$, $\Theta_{st}^{(j+p+1)} = 0$, $\Theta_{s,(t+p)}^{(j+p+1)} = 0$ when $t \leq$j+1, $\Theta_{(s+p),t}^{(j+p+1)} = 0$ when $s \leq$j+1, and $\Theta_{(s+p),(t+p)}^{(j+p+1)}$=0, when both $s \leq$j+1 and $t \leq$j+1. This finishes the proof for the two claims. A similar argument can also be applied to the multiple knockoffs. That is, if a same model is imposed on the original variables, then when applying Algorithm 2 to generate knockoffs, the conditional distribution is a normal one, and conditional on the nearby variants and their already constructed knockoffs, the jth variable is independent of the other original and knockoff variables.

In practice, as the model parameters are unknown, we use the following methods to estimate the model parameters, and approximately sample from the conditional distribution $\mathcal{L}\left(G_j \middle| G_{-j}, \tilde{G}_{1:(j-1)}\right)$ at each step:

1. Estimate the conditional mean by running a regression. Based on the first claim, the conditional mean should be a linear function of the nearby variants (those that belong to the same LD block) and their knockoffs, so we can estimate the conditional mean by regressing G_j on those variables. In this paper, we choose to include the variants in a nearby region (within 200kb), under the assumption that such a region is large enough to cover an LD block.
2. We did not estimate the conditional variance, instead, we permute the residuals as an approximation to sampling from the conditional distribution.

It is worthy to note that when the size of the blocks is large enough, we may think that such a model is a reasonable approximation to the true correlation structure of genetic variants. However, the larger the block, the higher the computational cost. To make a trade-off between the computational cost and model accuracy, in this paper we set the size of the block to be about 200kb (+/-100kb from the target variant), as the typical LD block is less than 100kb. Within these blocks LD decreases slowly with physical distance, but between blocks LD decays rapidly[1].

Proof of the exchangeability property of the sequential model for multiple knockoffs. Let $G = (G_1, \cdots, G_p)$ be p original explanatory variables (i.e. genetic variants in our case), also denoted as $\tilde{G}^0$; $\tilde{G}^{\mathrm{m}} = (\tilde{G}_1^m, \cdots, \tilde{G}_p^m)$, $1 \le m \le M$, be M $(M \ge 2)$ groups of knockoff features. $\sigma = (\sigma_j)_{1 \le j \le p}$ is a collection of p permutations over the set of integers $\{0, 1, \cdots, M\}$, with each σ_j corresponding to an original feature X_j. The variables after swapping are defined as $(\tilde{G}^0, \tilde{G}^1, \cdots, \tilde{G}^{\mathrm{M}})_{\mathrm{swap}(\sigma)} := (U^0, U^1, \cdots, U^{\mathrm{M}})$, with $U_j^{\mathrm{m}} = \tilde{G}_j^{\sigma_j(m)}$ for all $1 \le j \le p, 1 \le m \le M$. The extended exchangeability condition for multiple knockoffs is defined as follows.

Definition 1. The multiple knockoffs $(\tilde{G}^0, \tilde{G}^1, \cdots, \tilde{G}^M)$ *satisfy the extended exchangeability condition if* $(\tilde{G}^0, \tilde{G}^1, \cdots, G^M)_{swap(\sigma)}$ *follows the same distribution as* $(\tilde{G}^0, \tilde{G}^1, \cdots, \tilde{G}^M)$ *for any* σ.

We prove that if we generate multiple knockoffs by applying Algorithm 2, the extended exchangeability is satisfied. We denote the probability mass function (PMF) of $(G_{1:p}, \tilde{G}_{1:j-1}^1, \cdots, \tilde{G}_{1:j-1}^{\mathrm{M}})$ as $\mathcal{L}(G_{-j}, G_j, \tilde{G}_{1:j-1}^1, \cdots, \tilde{G}_{1:j-1}^{\mathrm{M}})$. Our argument is based on induction with the following induction hypothesis: after j steps, for all $1 \le l \le j$, the variables $(\tilde{G}_l^0, \tilde{G}_l^1, \cdots, \tilde{G}_l^M)$ are exchangeable with respect to any permutation σ_l over the set of integers $\{0, 1, \cdots, M\}$ in the joint distribution $\mathcal{L}(G_{1:p}, \tilde{G}_{1:j}^1, \cdots, \tilde{G}_{1:j}^M)$.

It is easy to check the induction hypothesis is true when $j = 1$. Next, assuming the induction hypothesis holds for the first $j - 1$, we show that it also holds after j steps. At step j, $\tilde{G}_j^1, \cdots, \tilde{G}_j^{\mathrm{M}}$ are conditionally independent and follow the same distribution. The conditional PMF of $\tilde{G}_j^{(1)}$ given $G_{1:p}, \tilde{G}_{1:j-1}^1, \cdots, \tilde{G}_{1:j-1}^{\mathrm{M}}$ is

$$\frac{\mathcal{L}(G_{-j}, \tilde{G}_j^1, \tilde{G}_{1:j-1}^1, \cdots, \tilde{G}_{1:j-1}^{\mathrm{M}})}{\sum_u \mathcal{L}(G_{-j}, u, \tilde{G}_{1:j-1}^1, \cdots, \tilde{G}_{1:j-1}^{\mathrm{M}})}.$$

Then the joint PMF of $(G_{1:p}, \tilde{G}_{1:j}^1, \cdots, \tilde{G}_{1:j}^{\mathrm{M}})$ is the product of the conditional PMF with the joint PMF of $(G_{1:p}, \tilde{G}_{1:j-1}^1, \cdots, \tilde{G}_{1:j-1}^{\mathrm{M}})$:

$$\frac{\prod_{m=0}^{M} \mathcal{L}(G_{-j}, \tilde{G}_j^{\mathrm{m}}, \tilde{G}_{1:j-1}^1, \cdots, \tilde{G}_{1:j-1}^{\mathrm{M}})}{\left(\sum_u \mathcal{L}(G_{-j}, u, \tilde{G}_{1:j-1}^1, \cdots, \tilde{G}_{1:j-1}^{\mathrm{M}})\right)^M}.$$

The PMF remains invariant with respect to any permutation of $(\tilde{G}_j^0, \tilde{G}_j^1, \cdots, \tilde{G}_j^{\mathrm{M}})$. Based on the induction hypothesis, we also have for any $l < j$, the joint distribution $\mathcal{L}$ is symmetric in $(\tilde{G}_l^0, \tilde{G}_l^1, \cdots, \tilde{G}_l^{\mathrm{M}})$. Combining these two facts, the induction hypothesis holds after j steps.

Proof of FDR control. For *KnockoffScreen*, we construct multiple groups of knockoff features and introduce a new feature importance statistic $\tau_{\Phi_{kl}} = T_{\Phi_{kl}}^{(0)} - \underset{1 \le m \le M}{\text{median}}\, T_{\Phi_{kl}}^{(m)}$ instead of $T_{\Phi_{kl}}^{(0)} - T_{\Phi_{kl}}^{(1)}$, where Φ_{kl} denotes a window on the genome. In this section, we show what with the newly introduced feature important statistic, the method still leads to valid FDR control.

Let $\Phi_{k_1 l_1}, \Phi_{k_2 l_2}, \cdots, \Phi_{k_W l_W}$ be a set of non-overlapping windows on the genome. Recall that each window $\Phi_{k_\omega l_\omega}$ $(1 \le \omega \le W)$ is defined to be $\Phi_{k_\omega l_\omega} = \{j: k_\omega \le j \le l_\omega\} \subset [p]$. In other words, $\Phi_{k_\omega l_\omega}$'s are disjoint subsets of $[p]$. $\mathcal{H}_0 = \{j: G_j \textit{ is a noncausal variant}\}$. For each window, we have introduced a pair of test statistics: $\tau_{\Phi_{k_\omega l_\omega}}$ and $\kappa_{\Phi_{k_\omega l_\omega}}$. Similar to all types of knockoff filters, the FDR control is achieved based on the following key property of the test statistics.

Property 1: conditioning on $\left(\tau_{\Phi_{k_\omega l_\omega}}\right)_{1 \le \omega \le W}$ and $\kappa_{\Phi_{k_\omega l_\omega}}$'s for non-null windows, $\kappa_{\Phi_{k_\omega l_\omega}}$ for null windows are i.i.d. random variables uniformly distributed on $\{0, 1, \cdots, M\}$.

Under multiple knockoffs framework, the property can be view as an extension of the sign-flipping one corresponding to the single knockoffs.

To show this property, we consider a collection of permutations $\left(\sigma_{\Phi_{k_\omega l_\omega}}\right)_{1 \le \omega \le W}$ on $\{0, 1, \cdots, M\}$ defined in the following way: if $\Phi_{k_\omega l_\omega} \subset \mathcal{H}_0$, then $\sigma_{\Phi_{k_\omega l_\omega}}$ can be any permutation; otherwise, $\sigma_{\Phi_{k_\omega l_\omega}}$ is the identity permutation. We will show the following two sets of random variables follow the same distribution:

$$\left(\left(\sigma_{\Phi_{k_\omega l_\omega}}\left(\kappa_{\Phi_{k_\omega l_\omega}}\right)\right)_{1 \le \omega \le W}, \left(\tau_{\Phi_{k_\omega l_\omega}}\right)_{1 \le \omega \le W}\right) \sim \left(\left(\kappa_{\Phi_{k_\omega l_\omega}}\right)_{1 \le \omega \le W}, \left(\tau_{\Phi_{k_\omega l_\omega}}\right)_{1 \le \omega \le W}\right)$$

The proof is based on the following observation: for window $\Phi_{k_\omega l_\omega}$ with test statistics $\tau_{\Phi_{k_\omega l_\omega}}$ and $\kappa_{\Phi_{k_\omega l_\omega}}$, if we apply any permutation $\sigma_{\Phi_{k_\omega l_\omega}}$ to variables and their knockoffs corresponding to the variants covered by the same window, then based on the permuted data set the test statistics are exactly $\tau_{\Phi_{k_\omega l_\omega}}$ and $\sigma_{\Phi_{k_\omega l_\omega}}\left(\kappa_{\Phi_{k_\omega l_\omega}}\right)$. More precisely, if we define $(\widetilde{\boldsymbol{G}}^{\mathbf{0}}, \widetilde{\boldsymbol{G}}^{\mathbf{1}}, \cdots, \widetilde{\boldsymbol{G}}^{\mathbf{M}})_{\text{swap}} := (\boldsymbol{U}^{\mathbf{0}}, \boldsymbol{U}^{\mathbf{1}}, \cdots, \boldsymbol{U}^{\mathbf{M}})$ as $\boldsymbol{U}_{\boldsymbol{j}}^{\mathbf{m}} = \widetilde{\boldsymbol{G}}_{\boldsymbol{j}}^{\boldsymbol{\sigma}_{\boldsymbol{\Phi}_{\boldsymbol{k_\omega l_\omega}}}(\boldsymbol{m})}$ for $j \in \Phi_{k_\omega l_\omega}$, and denote the test statistics for window $\Phi_{k_\omega l_\omega}$ based on $(\widetilde{\boldsymbol{G}}^{\mathbf{0}}, \widetilde{\boldsymbol{G}}^{\mathbf{1}}, \cdots, \widetilde{\boldsymbol{G}}^{\mathbf{M}})_{\text{swap}}$ as $\hat{\sigma}_{\Phi_{k_\omega l_\omega}}$ and $\hat{\tau}_{\Phi_{k_\omega l_\omega}}$, then $\hat{\tau}_{\Phi_{k_\omega l_\omega}} = \tau_{\Phi_{k_\omega l_\omega}}$ and $\hat{\sigma}_{\Phi_{k_\omega l_\omega}} = \sigma_{\Phi_{k_\omega l_\omega}}\left(\kappa_{\Phi_{k_\omega l_\omega}}\right)$. In combination with the fact that $(\widetilde{\boldsymbol{G}}^{\mathbf{0}}, \widetilde{\boldsymbol{G}}^{\mathbf{1}}, \cdots, \widetilde{\boldsymbol{G}}^{\mathbf{M}})_{\text{swap}}$ and $(\widetilde{\boldsymbol{G}}^{\mathbf{0}}, \widetilde{\boldsymbol{G}}^{\mathbf{1}}, \cdots, \widetilde{\boldsymbol{G}}^{\boldsymbol{M}})$ follow the same distribution, we have

$$\begin{aligned}
&\left(\left(\sigma_{\Phi_{k_\omega l_\omega}}\left(\kappa_{\Phi_{k_\omega l_\omega}}\right)\right)_{1 \le \omega \le W}, \left(\tau_{\Phi_{k_\omega l_\omega}}\right)_{1 \le \omega \le W}\right) \\
&= f\left((\widetilde{\boldsymbol{G}}^{\mathbf{0}}, \widetilde{\boldsymbol{G}}^{\mathbf{1}}, \cdots, \widetilde{\boldsymbol{G}}^{\mathbf{M}})_{\text{swap}}, \boldsymbol{Y}\right) \\
&\sim f\left((\widetilde{\boldsymbol{G}}^{\mathbf{0}}, \widetilde{\boldsymbol{G}}^{\mathbf{1}}, \cdots, \widetilde{\boldsymbol{G}}^{\boldsymbol{M}}), \boldsymbol{Y}\right) \\
&= \left(\left(\kappa_{\Phi_{k_\omega l_\omega}}\right)_{1 \le \omega \le W}, \left(\tau_{\Phi_{k_\omega l_\omega}}\right)_{1 \le \omega \le W}\right)
\end{aligned}$$

Here f is a function describing how we calculate test statistics based on the data. This finishes the proof for *Property 1*.

If *Property 1* holds, the knockoff filter is a special case of the Second Sequential Testing Procedure discussed by Barber and Candès[2], the FDR control can be obtained by using a similar argument as that used by Gimenez and Zou[3].

Supplementary Tables

Supplementary Table 1: Empirical evaluation of *KnockoffScreen* in the presence of population stratification driven by rare variants. Each cell presents the empirical FDR. γ quantifies the magnitude of population stratification; C: continuous trait; D: dichotomous trait. KnockoffScreen controls FDR at 0.10; Association Testing is based on the usual Bonferroni correction (0.05/number of tests), controlling FWER at 0.05 level.

γ	Trait	KnockoffScreen	KnockoffScreen 10 PCs	Association Testing	Association Testing 10 PCs
0	C	0.098	0.102	0.022	0.020
0.25	C	0.084	0.096	0.094	0.024
0.5	C	0.124	0.084	0.430	0.018
0.75	C	0.196	0.068	0.926	0.028
0	D	0.106	0.112	0.056	0.058
0.25	D	0.108	0.100	0.184	0.042
0.5	D	0.198	0.110	0.846	0.030
0.75	D	0.312	0.090	0.996	0.034

Supplementary Table 2: Tissue grouping of GenoNet scores. The GenoNet scores were trained using epigenetic annotations from the Roadmap Epigenomics Project across 127 tissues/cell types.

Epigenome ID (EID)	Standardized Epigenome name	Group
E062	Primary mononuclear cells fromperipheralblood	Blood
E034	Primary T cells fromperipheralblood	Blood
E045	Primary T cells e_ector/memory enriched from peripheral blood	Blood
E033	Primary T cells from cord blood	Blood
E044	Primary T regulatory cells fromperipheralblood	Blood
E043	Primary T helper cells fromperipheralblood	Blood
E039	Primary T helper naive cells fromperipheralblood	Blood
E041	Primary T helper cells PMA-I stimulated	Blood
E042	Primary T helper 17 cells PMA-I stimulated	Blood
E040	Primary T helper memory cells from peripheral blood 1	Blood
E037	Primary T helper memory cells from peripheral blood 2	Blood
E048	Primary T CD8+ memory cells from peripheral blood	Blood
E038	Primary T helper naive cells from peripheral blood	Blood
E047	Primary T CD8+ naive cells from peripheral blood	Blood
E029	Primary monocytes from peripheral blood	Blood
E031	Primary B cells from cord blood	Blood
E035	Primary hematopoietic stem cells	Blood
E051	Primary hematopoietic stem cells G-CSF-mobilized Male	Blood
E050	Primary hematopoietic stem cells G-CSF-mobilized Female	Blood
E036	Primary hematopoietic stem cells short term culture	Blood
E032	Primary B cells from peripheral blood	Blood

E046	Primary Natural Killer cells from peripheral blood	Blood
E030	Primary neutrophils from peripheral blood	Blood
E112	Thymus	Blood
E093	Fetal Thymus	Blood
E115	Dnd41 TCell Leukemia Cell Line	Blood
E116	GM12878 Lymphoblastoid Cells	Blood
E123	K562 Leukemia Cells	Blood
E124	Monocytes-CD14+ RO01746 Primary Cells	Blood
E071	Brain Hippocampus Middle	Brain
E074	Brain Substantia Nigra	Brain
E068	Brain Anterior Caudate	Brain
E069	Brain Cingulate Gyrus	Brain
E072	Brain Inferior Temporal Lobe	Brain
E067	Brain Angular Gyrus	Brain
E073	Brain Dorsolateral Prefrontal Cortex	Brain
E017	MR90 fetal lung _broblasts Cell Line	ConnectiveTissue
E026	Bone Marrow Derived Cultured Mesenchymal Stem Cells	ConnectiveTissue
E049	Mesenchymal Stem Cell Derived Chondrocyte Cultured Cells	ConnectiveTissue
E025	Adipose Derived Mesenchymal Stem Cell Cultured Cells	ConnectiveTissue
E023	Mesenchymal Stem Cell Derived Adipocyte Cultured Cells	ConnectiveTissue
E052	Muscle Satellite Cultured Cells	ConnectiveTissue
E055	Foreskin Fibroblast Primary Cells skin01	ConnectiveTissue
E056	Foreskin Fibroblast Primary Cells skin02	ConnectiveTissue
E057	Foreskin Keratinocyte Primary Cells skin02	ConnectiveTissue
E058	Foreskin Keratinocyte Primary Cells skin03	ConnectiveTissue
E028	Breast variant Human Mammary Epithelial Cells (vHMEC)	ConnectiveTissue
E114	A549 EtOH 0.02pct Lung Carcinoma Cell Line	ConnectiveTissue
E117	HeLa-S3 Cervical Carcinoma Cell Line	ConnectiveTissue
E119	HMEC Mammary Epithelial Primary Cells	ConnectiveTissue
E120	HSMM Skeletal Muscle Myoblasts Cells	ConnectiveTissue
E121	HSMM cell derived Skeletal Muscle Myotubes Cells	ConnectiveTissue
E122	HUVEC Umbilical Vein Endothelial Primary Cells	ConnectiveTissue
E125	NH-A Astrocytes Primary Cells	ConnectiveTissue
E126	NHDF-Ad Adult Dermal Fibroblast Primary Cells	ConnectiveTissue
E127	NHEK-Epidermal Keratinocyte Primary Cells	ConnectiveTissue
E128	NHLF Lung Fibroblast Primary Cells	ConnectiveTissue
E129	Osteoblast Primary Cells	ConnectiveTissue
E054	Ganglion Eminence derived primary cultured neurospheres	FetalBrain

E053	Cortex derived primary cultured neurospheres	FetalBrain
E070	Brain Germinal Matrix	FetalBrain
E082	Fetal Brain Female	FetalBrain
E081	Fetal Brain Male	FetalBrain
E013	hESC Derived CD56+ Mesoderm Cultured Cells	FetalTissue1
E005	H1 BMP4 Derived Trophoblast Cultured Cells	FetalTissue1
E006	H1 Derived Mesenchymal Stem Cells	FetalTissue1
E083	Fetal Heart	FetalTissue1
E099	Placenta Amnion	FetalTissue1
E089	Fetal Muscle Trunk	FetalTissue2
E090	Fetal Muscle Leg	FetalTissue2
E092	Fetal Stomach	FetalTissue2
E088	Fetal Lung	FetalTissue2
E080	Fetal Adrenal Gland	FetalTissue2
E091	Placenta	FetalTissue2
E085	Fetal Intestine Small	Gastrointestinal
E084	Fetal Intestine Large	Gastrointestinal
E109	Small Intestine	Gastrointestinal
E106	Sigmoid Colon	Gastrointestinal
E075	Colonic Mucosa	Gastrointestinal
E101	Rectal Mucosa Donor 29	Gastrointestinal
E102	Rectal Mucosa Donor 31	Gastrointestinal
E110	Stomach Mucosa	Gastrointestinal
E077	Duodenum Mucosa	Gastrointestinal
E066	Liver	Gastrointestinal
E118	HepG2 Hepatocellular Carcinoma Cell Line	Gastrointestinal
E059	Foreskin Melanocyte Primary Cells skin01	InternalOrgans
E061	Foreskin Melanocyte Primary Cells skin03	InternalOrgans
E027	Breast Myoepithelial Primary Cells	InternalOrgans
E100	Psoas Muscle	InternalOrgans
E104	Right Atrium	InternalOrgans
E095	Left Ventricle	InternalOrgans
E105	Right Ventricle	InternalOrgans
E065	Aorta	InternalOrgans
E079	Esophagus	InternalOrgans
E094	Gastric	InternalOrgans
E086	Fetal Kidney	InternalOrgans
E097	Ovary	InternalOrgans

E087	Pancreatic Islets	InternalOrgans
E098	Pancreas	InternalOrgans
E096	Lung	InternalOrgans
E113	Spleen	InternalOrgans
E063	Adipose Nuclei	Muscle
E108	Skeletal Muscle Female	Muscle
E107	Skeletal Muscle Male	Muscle
E078	Duodenum Smooth Muscle	Muscle
E076	Colon Smooth Muscle	Muscle
E103	Rectal Smooth Muscle	Muscle
E111	Stomach Smooth Muscle	Muscle
E002	ES-WA7 Cells	StemCell
E008	H9 Cells	StemCell
E001	ES-I3 Cells	StemCell
E015	HUES6 Cells	StemCell
E014	HUES48 Cells	StemCell
E016	HUES64 Cells	StemCell
E003	H1 Cells	StemCell
E024	ES-UCSF4 Cells	StemCell
E020	iPS-20b Cells	StemCell
E019	iPS-18 Cells	StemCell
E018	iPS-15b Cells	StemCell
E021	iPS DF 6.9 Cells	StemCell
E022	iPS DF 19.11 Cells	StemCell
E007	H1 Derived Neuronal Progenitor Cultured Cells	StemCell
E009	H9 Derived Neuronal Progenitor Cultured Cells	StemCell
E010	H9 Derived Neuron Cultured Cells	StemCell
E012	hESC Derived CD56+ Ectoderm Cultured Cells	StemCell
E011	hESC Derived CD184+ Endoderm Cultured Cells	StemCell
E004	H1 BMP4 Derived Mesendoderm Cultured Cells	StemCell

Supplementary Figures

Supplementary Figure 1 Distribution of power and false discovery proportion (FDP) at target FDR level 0.1 in simulation studies. The results are based on 1000 replicates and the same settings as in Figure 1.

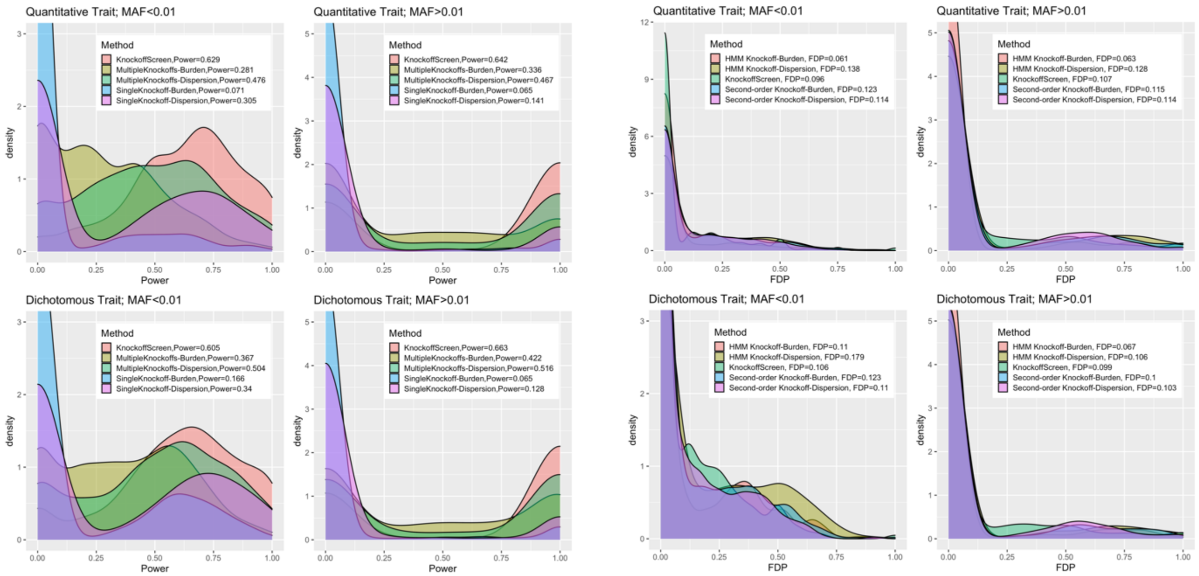

Supplementary Figure 2: Empirical validation of the extended exchangeability for rare variants. We generated 10,000 individuals with genetic data for a 200 kb region containing 1000 genetic variants, simulated using a coalescent model (COSI). To validate the extended exchangeability, we generated two knockoffs using the proposed algorithm and evaluated whether the second order (covariance between each pair of genetic variants) is exchangeable for both rare and common variants in the regions.

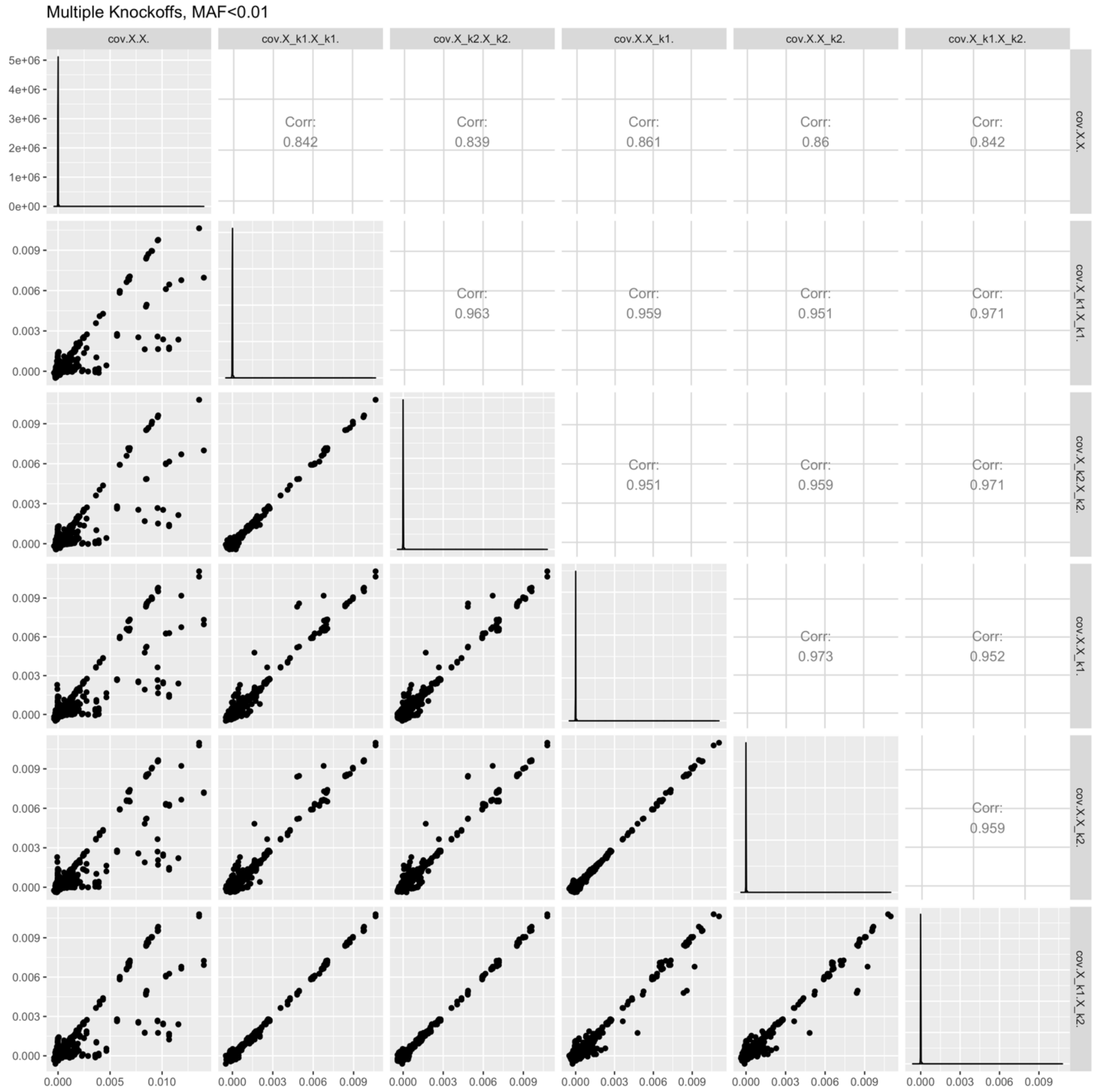

Supplementary Figure 3: Empirical validation of the extended exchangeability for common variants. We generated 10,000 individuals with genetic data for a 200 kb region containing 1000 genetic variants, simulated using a coalescent model (COSI). To validate the extended exchangeability, we generated two knockoffs using the proposed algorithm and evaluated whether the second order (covariance between each pair of genetic variants) is exchangeable for both rare and common variants in the regions.

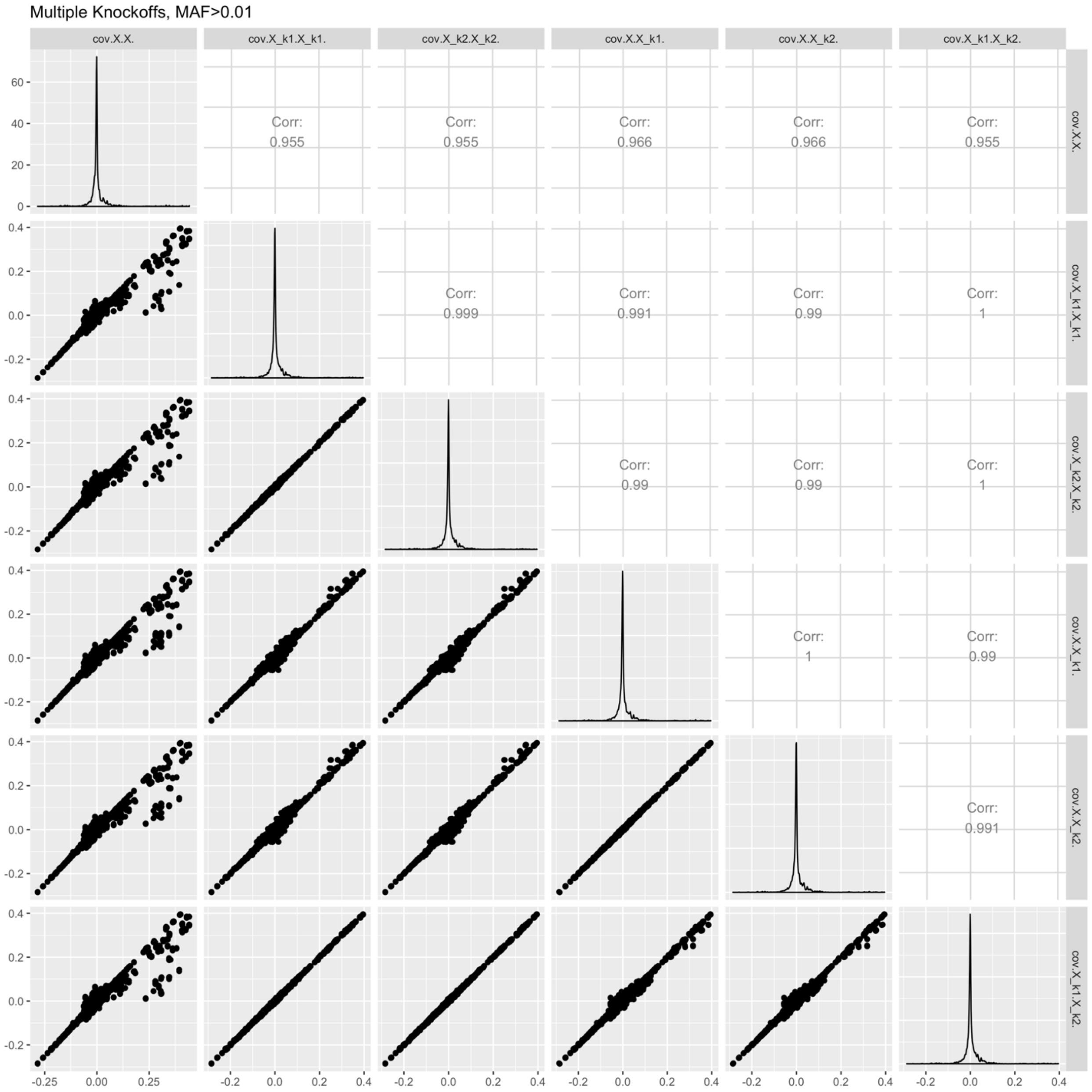

Supplementary Figure 4: *KnockoffScreen* application to the COPDGene study in TOPMed to identify variants associated with the FEV_1/FVC ratio in Non Hispanic White (NHW). The top-left panel presents the Manhattan plot of p-values from the conventional association testing with Bonferroni adjustment ($p < 0.05$/number of tested windows) for FWER control. The bottom-left panel presents the Manhattan plot of *KnockoffScreen* with target FDR at 0.1. The right panel presents a heatmap that shows stratified p-values of all loci passing the FDR=0.1 threshold, and the corresponding Q-values that already incorporate correction for multiple testing. The loci are shown in descending order of the knockoff statistics. For each locus, the p-values of the top associated single variant and/or window are shown indicating whether the signal comes from a single variant, a combined effect of common variants or a combined effect of rare variants. The names of those genes previously implicated by GWAS studies are shown in bold (names were just used to label the region and may not represent causative gene in the region).

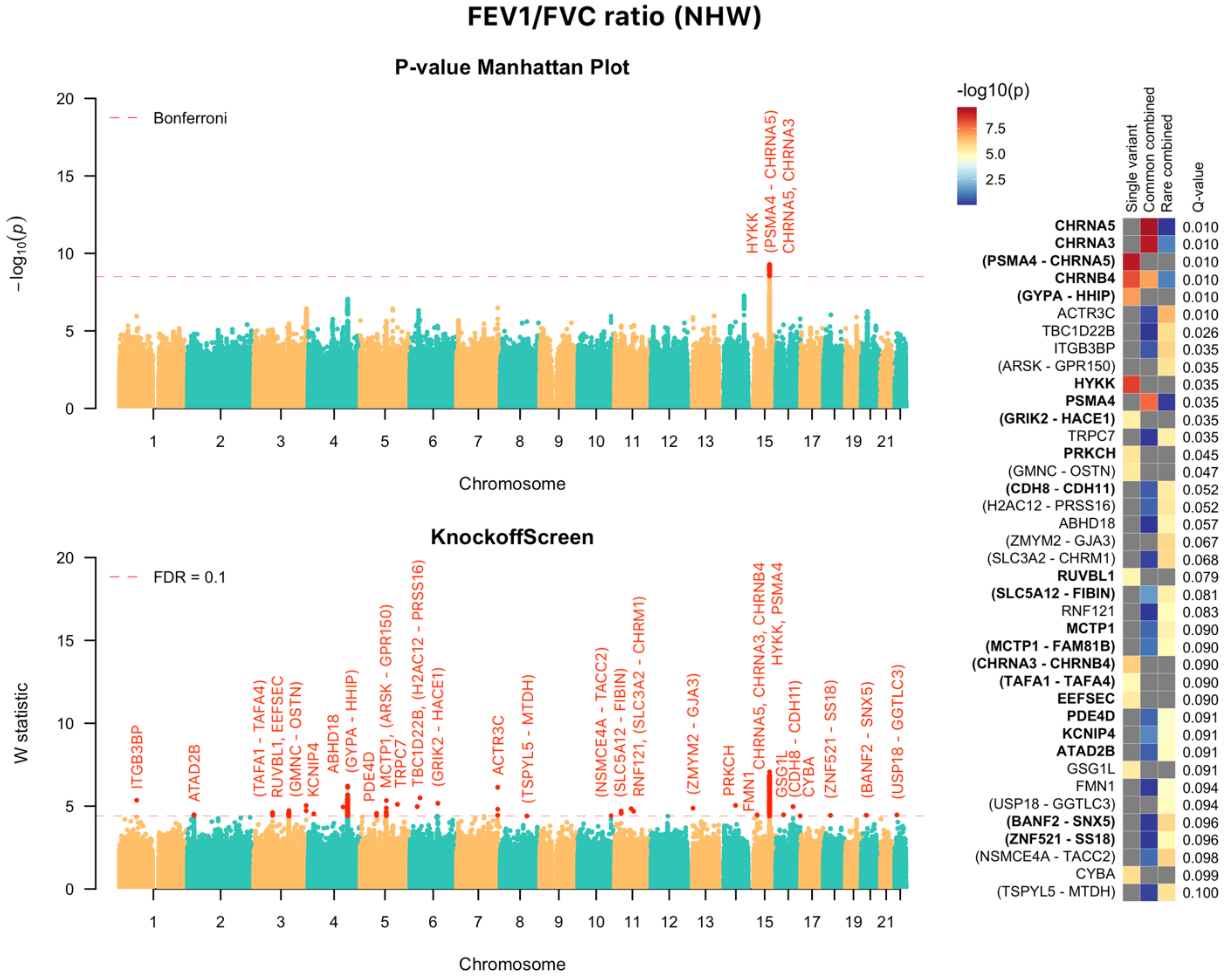

Supplementary Figure 5: QQ plots for all tests, common variants tests, and rare variant tests included in the *KnockoffScreen* procedure for all datasets used in the analyses.

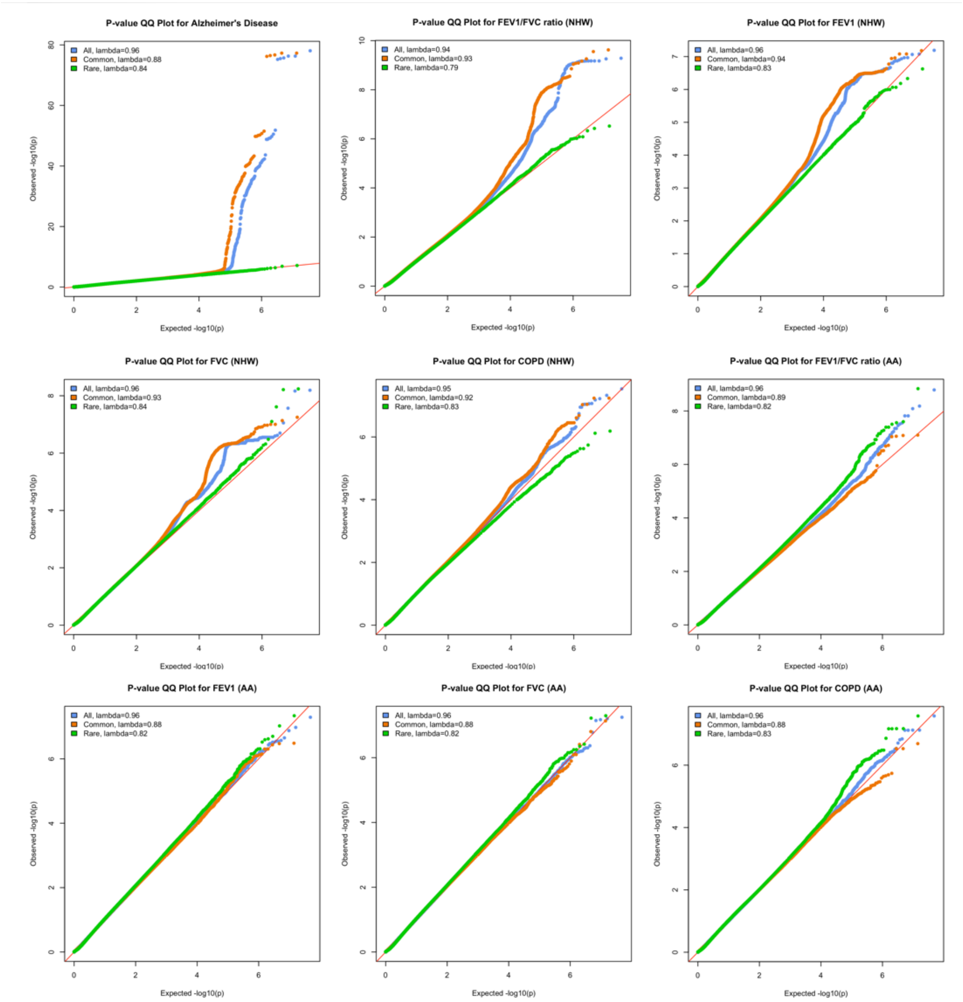

Supplementary Figure 6: The analysis of the ADSP and TOPMed data with the Benjamini–Hochberg procedure for FDR control. The left panel presents the Manhattan plot of adjusted p-values (Q-values; truncated at 10^{-10} for clear visualization) from the conventional association testing with the Benjamini–Hochberg adjustment for FDR control. The right panel presents a heatmap that shows stratified p-values (truncated at 10^{-10} for clear visualization) of all loci passing the FDR=0.1 threshold, and the corresponding adjusted p-values that already incorporate correction for multiple testing. For each locus, the adjusted p-values of the top associated single variant and/or window are shown indicating whether the signal comes from a single variant, a combined effect of common variants or a combined effect of rare variants. The names of those genes previously implicated by GWAS studies are shown in bold (names were just used to label the region and may not represent causative gene in the region).

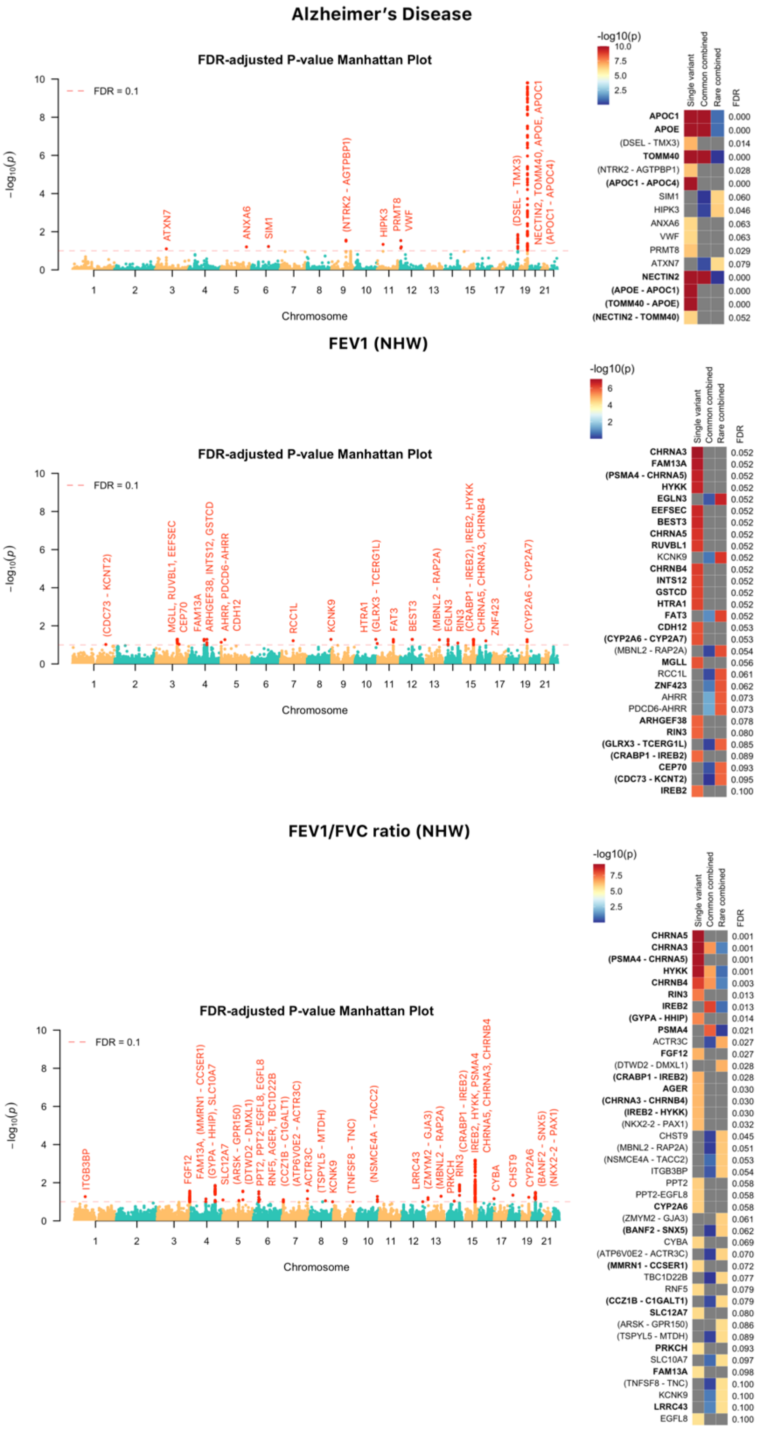

Supplementary Figure 7: Comparison with HMM (S=12) stratified by minor allele frequency. We generated 10,000 individuals with genetic data for a 200 kb region containing 1000 genetic variants, simulated using a coalescent model (COSI). We compared the proposed algorithm to HMM with number of states S=12 and evaluated whether the second order (correlation between each pair of genetic variants) is exchangeable. Each dot presents one variant/window. The left panels evaluate how the correlation structure of knockoffs is similar to that of the original variants; the right panels evaluate how the knockoffs preserve the correlation structure when one swaps a variant with its synthetic counterpart.

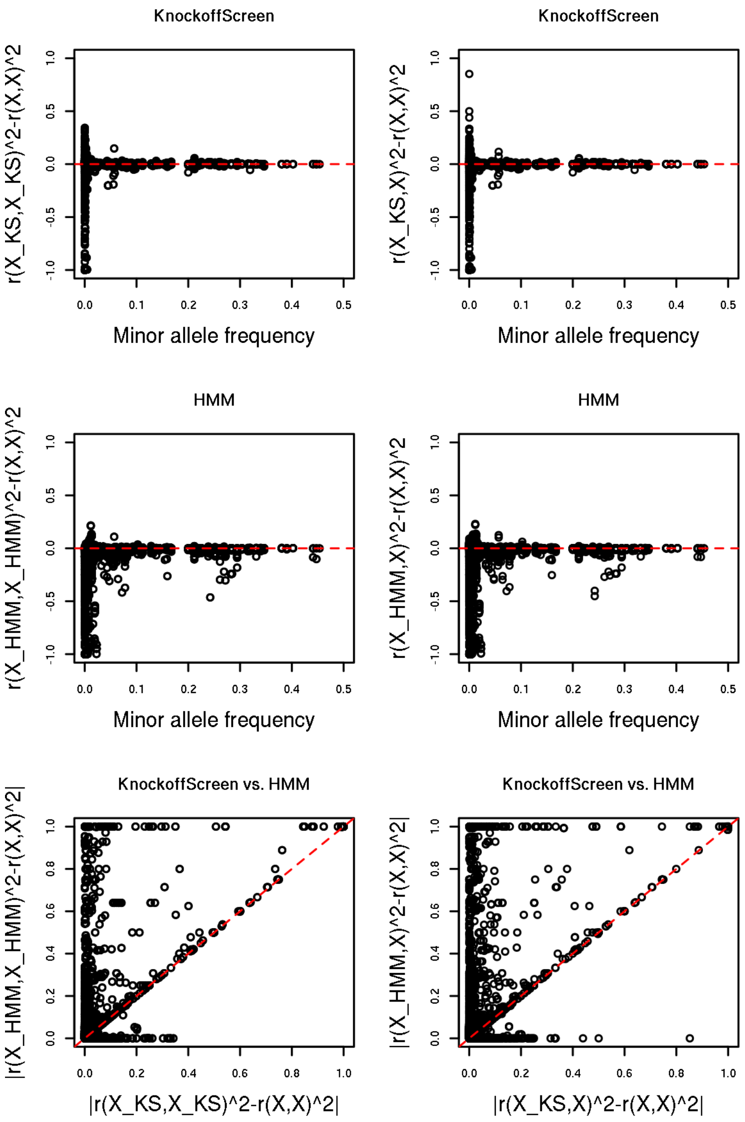

Supplementary Figure 8: Comparison with HMM (S=50) stratified by minor allele frequency. We generated 10,000 individuals with genetic data for a 200 kb region containing 1000 genetic variants, simulated using a coalescent model (COSI). We compared the proposed algorithm to HMM with number of states S=50 and evaluated whether the second order (correlation between each pair of genetic variants) is exchangeable. Each dot presents one variant/window. The left panels evaluate how the correlation structure of knockoffs is similar to that of the original variants; the right panels evaluate how the knockoffs preserve the correlation structure when one swaps a variant with its synthetic counterpart.

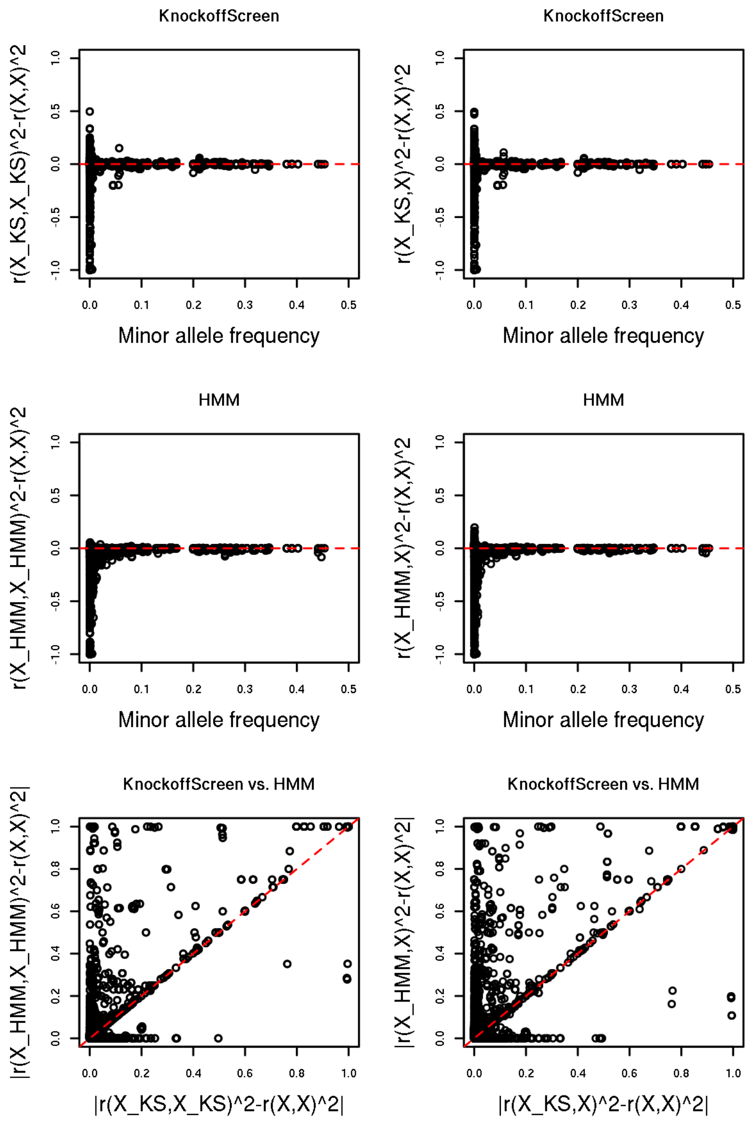

Supplementary References

1. Anderson, E.C. & Novembre, J. Finding haplotype block boundaries by using the minimum-description-length principle. *The American Journal of Human Genetics* **73**, 336-354 (2003).
2. Barber, R.F. & Candès, E.J. Controlling the false discovery rate via knockoffs. *The Annals of Statistics* **43**, 2055-2085 (2015).
3. Gimenez, J.R. & Zou, J. Improving the Stability of the Knockoff Procedure: Multiple Simultaneous Knockoffs and Entropy Maximization. *arXiv preprint arXiv:1810.11378* (2018).
